# Analgesic effects of a hydro-ethanolic whole plant extract of *Synedrella nodiflora* (L.) Gaertn in paclitaxel-induced neuropathic pain in rats

**DOI:** 10.1186/s13104-017-2551-7

**Published:** 2017-06-26

**Authors:** Patrick Amoateng, Samuel Adjei, Dorcas Osei-Safo, Kennedy Kwami Edem Kukuia, Irene Akwo Kretchy, Joseph Adusei Sarkodie, Benoit Banga N’Guessan

**Affiliations:** 1 Department of Pharmacology & Toxicology, School of Pharmacy, College of Health Sciences, University of Ghana, P. O. Box LG 43, Legon, Accra, Ghana; 2 Department of Animal Experimentation, Noguchi Memorial Institute for Medical Research, College of Health Sciences, University of Ghana, P. O Box LG 581, Legon, Accra, Ghana; 3 Department of Chemistry, School of Physical and Mathematical Sciences, College of Basic and Applied Sciences, University of Ghana, P. O. Box LG 56, Legon, Accra, Ghana; 4 Department of Pharmacy Practice & Clinical Pharmacy, School of Pharmacy, College of Health Sciences, University of Ghana, P. O. Box LG 43, Legon, Accra, Ghana; 5 Department of Pharmacognosy & Herbal Medicine, School of Pharmacy, College of Health Sciences, University of Ghana, P. O. Box LG 43, Legon, Accra, Ghana

**Keywords:** *Synedrella nodiflora*, Pregabalin, Paclitaxel, Neuropathic, Pain

## Abstract

**Background:**

*Synedrella nodiflora* is used by traditional healers in Ghana for the management of epilepsy and pain. The hydro-ethanolic extract of the whole plant has demonstrated antinociceptive effect in various animal models of pain. This study investigated the potential benefit of the hydro-ethanolic extract in a rat model of paclitaxel-induced neuropathic pain.

**Methods:**

Neuropathy was induced in rats by a continuous intraperitoneal administration of paclitaxel (2 mg/kg) for 5 days. Baseline latencies to thermal pain were recorded before the first injection of paclitaxel and during the 5 day induction period. Following the induction, the rats in designated treatment group were treated with the hydro-ethanolic extract (100, 300 and 1000 mg/kg, p.o) or pregabalin (10, 30 and 100 mg/kg) or vehicle (distilled water) and their responses to thermal hyperalgesia measured every 30 for a total period of 3 h.

**Results:**

There was a significant difference between the baseline reaction latency and what was observed on the 5th day of the induction of neuropathy. Two days after the induction of neuropathy, the extract and pregabalin significantly and dose-dependently produced antinociceptive effect during the 3-h test period.

**Conclusion:**

The hydro-ethanolic extract of the whole plant of *Synedrella nodiflora* possess analgesic effect in paclitaxel-induced neuropathy in rats.

## Background

Chemotherapy-induced peripheral neuropathy (CIPN) is a disabling adverse reaction of quite a number of commonly used anticancer agents such as paclitaxel, vincristine and cisplatin [1, 2]. The incidence and severity of paclitaxel-induced neuropathy is associated with increasing cumulative doses of paclitaxel [3, 4]. Paclitaxel, a derivative of the Pacific yew tree (*Taxus brevifolia*), is usually used in the treatment of cancers of the cervix, breasts, lungs, head and neck [4, 5]. Peripheral sensory neuropathy involving numbness and tingling in the extremities, often accompanied by burning pain is a common side effect associated with the use of paclitaxel [6–8]. Treatment of CIPN is often difficult and it has been estimated that only 40–60% of patients receiving therapy with conventional treatments including opioid analgesics, anticonvulsants and tricyclic antidepressants achieve partial relief [9, 10]. Optimized treatment regimen in most patients with complicated neuropathic pain conditions includes a combination of analgesics from multiple drug classes to address multiple pain mechanisms and this presents with a myriad of unwanted side effects.

In developing countries and some developed countries, the use of plants and plant-based therapies are gradually becoming the alternative sources of medicines for orthodox drug-resistant diseases. These agents are usually the only remedy in areas where access to primary health care is difficult or expensive. Several plants and isolated compounds being tested against paclitaxel-induced neuropathic pain have proven useful with alternate drugs being developed [11–15].

*Synedrella nodiflora* (L.) Gaertn (family Asteraceae) is a common tropical weed which grows along the banks of rivers and often under shades of trees [16]. In Ghana, traditional healers boil the whole plant and the aqueous extract drunk for the treatment of epilepsy and pain [16]. The hydro-ethanolic extract of the whole plant has demonstrated anticonvulsant, sedative, in vitro antioxidant and free radical scavenging properties, and anti-nociceptive properties in several murine models of acute and chronic pain [17–20]. The present study was conducted to provide additional pharmacological information regarding the analgesic effect of the hydro-ethanolic extract of the whole plant of *Synedrella nodiflora* in painful paclitaxel-induced peripheral neuropathy.

## Results

### Qualitative phytochemistry of SNE

S*ynedrella nodiflora* extract contained flavonoids, tannins, saponins, alkaloids, cardiac glycosides, coumarins, triterpenes, sterols, anthraquinones and phenolic compounds.

### Induction of neuropathy

**Fig. 1:**
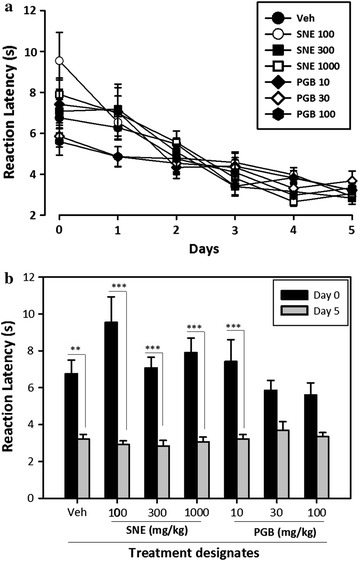
The effect of a daily intraperitoneal injection of paclitaxel (2 mg/kg) on the reaction latency (s) in rats for 5 days. **a** A time-course event from day 0, 1, 2, 3, 4 and 5. **b** A comparison of reaction latency on day 0 and day 5. Data are mean ± SEM (n = 5). **p ≤ 0.01 and ***p ≤ 0.001 compared to vehicle group (two-way ANOVA followed by a Bonferroni’s post-hoc test)

Clinical observation of the rats during the 5-day induction of neuropathy with paclitaxel revealed the absence of any ill-health such as alopecia, diarrhoea or weight loss. An assessment of the reaction latencies to thermal pain during the induction period yielded a gradual decline of the latencies from day 0 to day 5. There was no significant difference between the various designated groups during the induction period (P = 0.9416, F_6,35_ = 0.2821, Fig. 1a). However there was significant decline of the paw withdrawal latencies from day 0 to day 5 suggesting a reduced pain threshold and an induction of peripheral neuropathy by day 5 (P = 0.02; a two-way ANOVA, Fig. 1b).

### Effect of SNE and pregabalin on thermal hyperalgesia after the paclitaxel-induced neuropathy

**Fig. 2:**
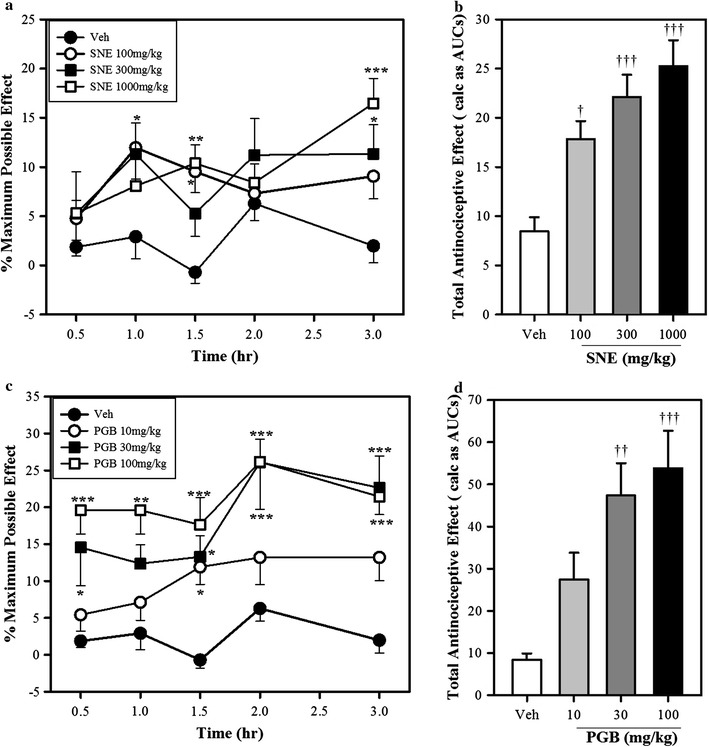
The effect of SNE (100–1000 mg/kg, p.o) and PGB (10–100 mg/kg, p.o) on thermal hyperalgesia in paclitaxel-induced neuropathy in rats. The *left panels* represent a time-course effects of SNE (**a**) and PGB (**c**) on day 8 after the induction of the neuropathy. The *right panels* also represent the total antinociceptive effects (calculated from the AUCs) of SNE (**b**) and PGB (**d**). Data are mean ± SEM (n = 5). *P ≤ 0.05, **P ≤ 0.01, and ***P ≤ 0.001 compared to vehicle group (two-way ANOVA followed by a Bonferroni’s posthoc test). ^†^P ≤ 0.05, ^††^P ≤ 0.01 and ^†††^P ≤ 0.001 compared to vehicle group (one-way ANOVA followed by a Dunnett’s multiple comparison test)

*Synedrella nodiflora* extract produced an overall significant increase in the thermal pain threshold of the SNE-treated rats in comparison to the vehicle-treated group of rats during the 3-h test period (P = 0.01, F_3,16_ = 55.28, one-way ANOVA, Fig. 2a). The analgesic effect of SNE was significant for dose level 300 mg/kg at 1 h and for dose levels 100 and 1000 mg/kg at 1.5 h post-treatment. However at 2 h post-treatment, none of the doses of SNE produced any significant effect and at the third hour, the 300 and 1000 mg/kg were significantly analgesic (A two-way ANOVA, Fig. 2a). Analysis of the AUCs of SNE demonstrates significant dose-dependent analgesic effect (P = 0.0002 F_3, 15_ = 12.47, One-way ANOVA, Fig. 2b).

Pregabalin, on day 8, significantly increased the thermal pain threshold over the 3-h test period (P < 0.0001, F_3,16_ = 19.88, one-way ANOVA, Fig. 2c). The analgesic effect was significant for dose levels 30 and 100 mg/kg, 30 min post-treatment and at 100 mg/kg, the effect was consistently significant at all times when measured up to the third hour. Additionally, dose level 30 mg/kg produced significant analgesic effect on the 1.5, 2 and 3 h intervals of the test period. A significant analgesic effect of the dose level 10 mg/kg was observed at time 1.5 h post-treatment (two-way ANOVA, Fig. 2c). The total anti-nociceptive effect was significant and dose-dependent (P = 0.0008, F_3,16_ = 9.39, one-way ANOVA, Fig. 2d).

**Fig. 3:**
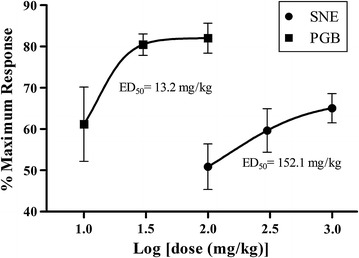
Dose-response curves of SNE (100–1000 mg/kg, p.o) and PGB (10–100 mg/kg, p.o) on thermal hyperalgesia 2 days-post paclitaxel-induced neuropathy in rats (day 8)

The ED_50_ (mg/kg) of SNE and pregabalin was 152.1 and 13.2 respectively, thus SNE is about 11 times less potent than pregabalin (Fig. 3).

## Discussion

The study described demonstrates that a hydro-ethanolic extract of the whole plant of *Synedrella nodiflora* (SNE) possesses potent analgesic effect in a paclitaxel-induced peripheral neuropathy in rats. This finding confirms earlier reports of the analgesic effect of this extract in various animal models of pain [17, 19].

A 5-day continuous intraperitoneal injection of paclitaxel into rats produced peripheral neuropathy demonstrated by a fall in thermal pain threshold from day 0 to day 5 (Fig. 1). Though the exact mechanism by which paclitaxel causes peripheral neuropathy is yet to be elucidated, current findings suggest that paclitaxel may produce nerve damage by disrupting the action of microtubules needed for axonal transport [24, 25]. Paclitaxel is also known to cause swelling of mitochondria in axons leading to loss of cellular functions and subsequent alteration of intracellular calcium levels in the axons and an initiation of apoptosis [26–29]. The ability of the extract to ameliorate the thermal hyperalgesia 2-days post induction of neuropathy corroborates its role in the management of paclitaxel-induced neuropathic pain [17]. The exact mechanism by which SNE attenuates thermal hyperalgesia in this model of peripheral neuropathy is not known and is currently the subject of concern for future work. However, an earlier report suggests that SNE’s analgesic effect is centrally mediated and may be associated with purinergic pathways [19]. This may serve as a starting point for the future elucidation of the anti-neuropathic pain mechanism of SNE. Another area to consider in elucidating the mechanism of action of SNE, is to investigate the effect of the extract on glutamatergic neurotransmission or NMDA receptor activation [30], since this neurotransmitter system and its associated NMDA receptors are implicated in paclitaxel-induced neuropathic pain [31–34].

Qualitatively, flavonoids, tannins, saponins, alkaloids, cardiac glycosides, coumarins, triterpenes, sterols, anthraquinones and phenolic compounds were detected in SNE. Some of these classes of compounds are known to have analgesic properties in various models of pain [35]. Thus, the analgesic effect of SNE in neuropathy could be attributed to the presence of these phytochemical constituents. Future research can also focus on isolating and characterizing the active principle(s) responsible for this activity.

Pregabalin is used clinically in the pharmacologic management of CIPN and this effect has been found to exist experimentally [36, 37]. The analgesic and anti-epileptic actions of pregabalin are associated with its antagonistic effect on α_2_–δ1 Ca^2+^ channel subunit of N-type voltage dependent calcium channels [37]. This contributes to the reduction of excitatory neurotransmitters released, the inhibition of synaptic transmission and other cellular enzymatic cascade reactions that lead to pain sensation [37, 38]. Thus, pregabalin’s potent analgesic effect in the paclitaxel-induced neuropathy in rats as demonstrated in this study confirms previous reports [39, 40].

## Conclusion

The hydro-ethanolic extract of *Synedrella nodiflora* possess analgesic activity against paclitaxel-induced thermal hyperalgesia in rats.

## Methods

### Drugs and chemicals

Pregabalin (Lyrica^®^) was purchased from Pfizer Pharmaceuticals, New York, USA and paclitaxel (Taxol^®^) from Bristol-Myers-Squibb, New York, USA.

### Plant collection and extraction

Samples of the plant were collected from the Botanical Gardens, University of Ghana, Legon, Accra in August 2012 and the samples were identified and authenticated at Ghana Herbarium, Department of Botany, University of Ghana, a where a voucher specimen (PA01/UGSOP/GH12) was kept. The hydro-ethanolic extract was prepared as previously described [18]. Briefly, the samples of the collected plant were air-dried for 7 days and powdered. Two kilogram of the powder were cold-macerated with 70% v/v of ethanol in water. The hydro-ethanolic extract was then evaporated using a rotary evaporator (Buchi Rotavapor^®^ R-300, Flawil, Switzerland) under reduced pressure to remove ethanol. The aqueous portion was frozen at −20 °C and lyophilized (Bench-top Freeze Dryer, Labfreez Instruments Co., Ltd, Beijing, China). A 7% yield was obtained and the dried sample (SNE) was kept in a dessicator.

### Qualitative phytochemistry of SNE

*Synedrella nodiflora* extract was screened for the presence of flavonoids, tannins, saponins, alkaloids, cardiac glycosides, coumarins, triterpenes, sterols, anthraquinones and phenolic compounds using standard protocols [21].

### Experimental animals and housing

Male Sprague–Dawley rats (Hsd:SD strain), weighing 150–200 g and 6–8 weeks old, were obtained from and maintained at the Department of Animal Experimentation, Noguchi Memorial Institute for Medical Research (NMIMR), University of Ghana, Legon, where all experimental procedures were performed. All animal procedures and techniques used in these studies were approved by the Scientific and Technical Committee (STC) of the Noguchi Memorial Institute for Medical Research [reference number STC-6 (1) 2012–13] and also by the Noguchi Institutional Animal Care and Use Committee (NIACUC), College of Health Sciences, University of Ghana with protocol number NIACUC-2012-01-1E. The animals were housed in groups of five in stainless steel cages (34 cm × 47 cm × 18 cm) with soft wood shavings as bedding and maintained under laboratory conditions (temperature 22 ± 2 °C, relative humidity 60–70%, and 12 h light–dark cycle). Additionally, the animals were fed with normal commercial pellet diet (AGRIMAT, Kumasi), and given water ad libitum. All experiments were performed during the day between the hours of 8:00–15:00 GMT.

### Induction of peripheral neuropathy with paclitaxel

Paclitaxel-induced neuropathy in Sprague–Dawley rats were performed as previously described [21]. Briefly, an intraperitoneal injection of 2 mg/kg of paclitaxel was administered to the rats daily for 5 days. Baseline measurements of the reaction latency of paw withdrawals or vocalizations indicative of pain were measured 30 min after the injection of the paclitaxel using the hotplate test.

### Extract/drug treatment of paclitaxel-induced neuropathic pain

Two days after the induction of neuropathy with paclitaxel (day 8), the rats were treated with SNE (100–1000 mg/kg, p.o), pregabalin (10–100 mg/kg, p.o) or vehicle (distilled water). Pregabalin was used as the reference drug because it has both anticonvulsant and analgesic effect in neuropathic pain. The extract also has demonstrated similar pharmacological characteristics being anticonvulsant and analgesic [18, 19].

### Behavioural assessment of neuropathic pain

Thermal hyperalgesic test was performed in the rats after the paclitaxel-induced neuropathy using the hotplate test as previously described [22, 23]. Thirty minutes prior to this test, the animals in the designated treatment groups received the extract (100, 300 or 1000 mg/kg, p.o) or pregabalin (10, 30 or 100 mg/kg p.o) or distilled water (0.01 ml/kg). Then, the animals were gently dropped onto the hot plate (Socrel Hot-plate Model DS37; Ugo Basile, Comerio VA, Italy) that was preheated to 55 °C and cut-off latency of 20 s was used. Paw withdrawal latency was recorded using a timer that was started when the animal was released onto the preheated plate and stopped at the moment of withdrawal, shaking, or licking of the hind paws and this time recorded as the reaction latency. The determination was repeated every 30 min for a total period of 3 h. Percentage maximum possible effects (% MPE) for each animal’s response was calculated based on the formula:

$$\% \,{\text{MPE = }}\frac{{{\text{Post-drug latency}}\; - \;{\text{Pre-drug latency}}}}{{{\text{Cut-off latency}}\; - \;{\text{Pre-drug latency}}}} \times 100$$

ED_50_ values were determined for the extract and reference drug to compare their potencies.

## Statistical analysis

GraphPad Prism Version 5.0 for Windows was used for all statistical analyses and ED_50_ determination. A *P* ≤ 0.05 was considered statistically significant in all analysis (one or two-way ANOVA in comparison to vehicle-treated groups and followed by an appropriate posthoc test). The graphs were plotted using Sigma Plot for Windows Version 11.0 (Systat Software Inc., Germany).

## Abbreviations

ANOVA: analysis of variance
CIPN: chemotherapy-induced peripheral neuropathy
ED_50_: effective dose at 50%
NIACUC: Noguchi Institutional Animal Care and Use Committee
NMDA: *N*-methyl-d-aspartic acid
NMIMR: Noguchi Memorial Institute for Medical Research
SNE: *Synedrella nodiflora* extract
STC: Scientific and Technical Committee
